# Comparative evaluation of the efficacy of customized maxillary oral appliance with mandibular advancement appliance as a treatment modality for moderate obstructive sleep apnea patients—a randomized controlled trial

**DOI:** 10.1186/s13063-022-07054-6

**Published:** 2023-02-01

**Authors:** Vikram Belkhode, Surekha Godbole, Sharayu Nimonkar, Sweta Pisulkar, Pranali Nimonkar

**Affiliations:** 1 Sharad Pawar Dental College and Hospital, Datta Meghe Institute of Medical Sciences, Deemed to be University, Sawangi, Wardha, Maharashtra India; 2 Department of Prosthodontics, Sharad Pawar Dental College and Hospital, Datta Meghe Institute of Higher Education and Research, Wardha, Wardha, India; 3 Department of Prosthodontics, Sharad Pawar Dental College & Hospital, Datta Meghe Institute of Medical Sciences (Deemed to be University), Sawangi (Meghe), Wardha, Maharashtra India; 4 New SBI Colony NisargNagri, Nagpur Road, Wardha, 442001 India; 5 Trauma Care Centre, Government Medical College, Nagpur, Maharashtra India

**Keywords:** Apnea/hypopnea index, Customized or custom-made maxillary oral appliance, Mandibular advancement splint, Polysomnography, Obstructive sleep apnea disorder

## Abstract

**Background:**

Obstructive sleep apnea (OSA) is quite common among the adult population, according to recent epidemiological studies. The most frequently suggested alternate treatment for mild to moderate OSA is oral appliances (OA). The purpose of the present study was to assess as well as compare the effectiveness of custom-made maxillary oral appliances against mandibular advancement appliances in the care of individuals suffering from moderate obstructive sleep apnea.

**Methods:**

A prospective interventional research was carried out with 40 participants. Polysomnography (PSG) was done and the participants with an apnea-hypopnea index (AHI) >15–30 were involved in the research. Study participants were randomly split up into two test groups: group I was the “Control Group” (group treated with a mandibular advancement device (MAD), *n*=20), while group II was exposed to a “customized maxillary oral appliance” (CMOA, *n*=20). Both groups had reference measures for AHI, blood oxygen saturation (SpO2), oro-nasal airflow via respiratory disturbance index (RDI), and the Epworth Sleepiness Scale (ESS). Appliances were fabricated and delivered to the respective study group participants. PSG was again conducted after a period of 1 and 3 months of appliance delivery and re-evaluation was done for all the parameters and was compared with reference measurements. The facts were analyzed using descriptive and analytical statistical methods. The statistical program utilized in the study was “SPSS (Statistical Package for Social Sciences) Version 20.1.” After 1 and 3 months, the statistical significance between the two study groups was assessed at *P*<0.05.

**Results:**

The analysis of mean AHI, SPO2, RDI, and ESS for both test groups manifested statistically significant measures (*P*<0.001). The study results revealed a statistically significant depletion in mean AHI scores, improvement in mean SPO2 scores, and reduction in mean RDI scores and ESS scores when compared with reference measurements to 1 month, 1 to 3 months, and between reference measurements and 3 months.

**Conclusion:**

The CMOA was effective in managing moderate OSA and has great therapeutic potential. It can be an option for the MAD for treating patients suffering from moderate obstructive sleep apnea.

**Trial registration:**

The study was registered under Clinical Trials Registry-India and the registration number is CTRI/2020/07/026936. Registered on 31 July 2020

## Background

Obstructive sleep apnea (OSA) is a condition in which the upper airway is partially or completely blocked during sleep, resulting in arterial oxygen desaturation and arousals. Excessive daytime sleepiness, cognitive problems, obesity, type 2 diabetes mellitus, hypertension, exacerbation of the chronic obstructive pulmonary disease, apnea, nocturnal awakening, episodes of choking during sleep, morning headache, and other manifestations and co-morbidities are all linked to OSA [1–3]. Severe OSA is a significant danger of developing atherosclerosis, sudden myocardial infarction, and overall mortality [4].

OSA has also been found to be a self-sustaining possibility in developing cardiovascular disease, ischemic stroke, and general mortality. The patients suffering from OSA have reported poor quality of life and also have found notable increased events of road traffic accidents [5].

Polysomnography is used to diagnose OSA (PSG). PSG uses the “Apnea/Hypopnea Index (AHI)” to assess the severity of OSA. Based on the AHI score, OSA is characterized as mild (AHI 5–15), moderate (AHI 15–30), or severe (AHI >30) [6].

Several methods for treating OSA have been well-documented in the literature. The most common among them are behavioral and surgical weight loss therapies, positional therapy, pharmacological therapy, surgical therapies (pharyngeal and maxillomandibular surgeries), continuous positive airway pressure (CPAP), and oral appliances (OA) such as the mandibular advancement device (MAD) [7–9]. Among all the listed non-surgical treatment options, only CPAP and OA are highly satisfactory. CPAP therapy is considered to be a gold standard treatment option for people with OSA and is universally approved. CPAP, on the other hand, has a slew of drawbacks, including muscle sagging, discomfort with pressure sensation and leakage, skin inflammation, machine noise, and a slew of other issues that make it unsuitable for users [10–12].

MAD has emerged as a feasible, plausible replacement, and the most accepted and chosen therapy for mild to moderate OSA patients. Many authors have confirmed the role of MAD in lowering the AHI episodes and enhancing the quality of life among the individuals suffering from it, in comparison to CPAP in their study publications [13, 14]. The working principle of MAD is by clasping the lower jaw in an advanced and descending position which enlarges the upper airway space and substantially reduces the AHI [15].

However, various side effects of MAD have been observed in several long-term model analysis studies, including dental pain, temporomandibular joint issues, xerostomia or excess salivation, and gum irritation [16].

Current analysis has suggested that 936 million individuals globally are suffering from OSA. Considering the numerical values given by the World Health Organization indicating the elevated occurrence of OSA in the general public and the complexities and complications of the existing devices used in managing OSA has resulted in a need of introducing a new effective treatment modality in this field. The customized maxillary oral appliance (CMOA) is an oral appliance designed to be anchored on the maxillary arch at an increased vertical dimension of 2mm that facilitates the advanced and descending position of the lower jaw which results in an enlarged upper airway. To treat OSA, it employs the principles of mandibular advancement splint and tongue holding appliance. CMOA increases the vertical within the limits at present occlusion and hence the chances of changes in the dentition are eliminated as seen in MAD [17]. Because MAD is the most widely used oral appliance for treating OSA, it is employed in this study as a “Control Group” to assess the efficiency of the newly created customized maxillary oral appliance to it. The null hypothesis of the current study was that there is no difference between customized maxillary oral appliances and the mandibular advancement device in the effects of the treatment for moderate OSA.

By comparing its efficacy to MAD, the current study intends to introduce this new oral appliance, CMOA, in controlling OSA as a unique remedial choice for individuals suffering from mild OSA.

## Materials and methods

### Source of data

This study volunteered patients from both genders with the age ranging from 30 to 50 years to turn up in the Sleep Medicine Department of AVBRH and JNMC, Wardha, who was diagnosed with cases of moderate OSA. The duration of the study ranged from June 2020 to May 2021.

### Ethical aspects

The study received approval from the Institutional Ethical Committee (Ref. no- DMIMS(DU)/IEC/2020-21/8811). The study was filed as a randomized controlled trial after receiving approval (CTRI/2020/07/026936). Before the study began, the participants were informed about the study and signed informed permission forms were filled them.

### Study design (Fig. 1)

This study was a two-armed (MAD and CMOA) randomized, controlled, parallel, double-blind clinical investigation.

**Fig. 1:**
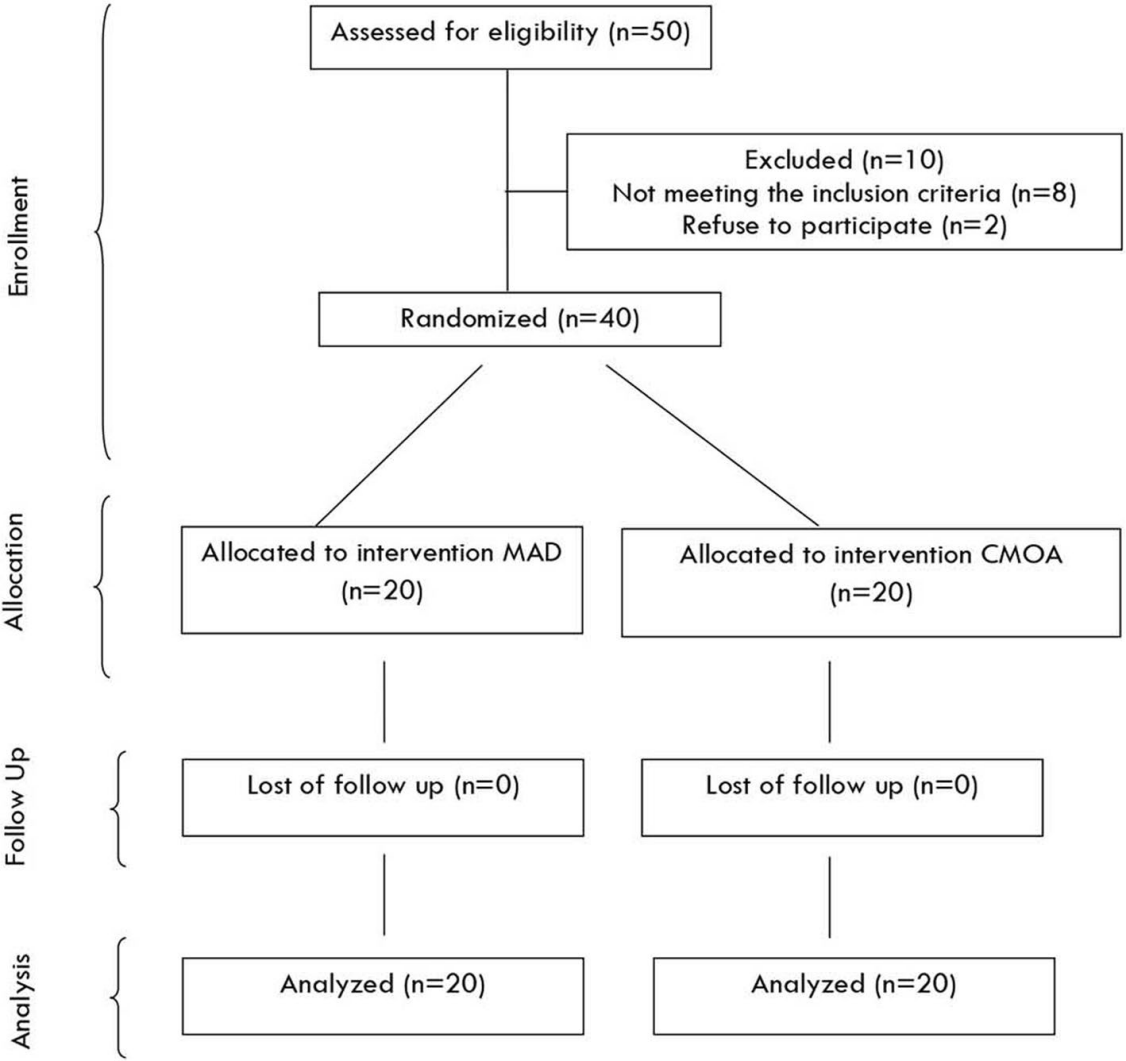
CONSORT flow diagram for randomization

### Sample size calculation

The software used for sample size calculation was N Master V.2.0. The sample size of the study was 40. The minimal sample size computed for each group based on the study’s 80% power was 16. However, 20 samples were chosen from each group to reduce mistakes and to account for any instances lost during follow-up. The following formula was applied for sample size calculation:

$${\displaystyle \begin{array}{l}k=\frac{n_2}{n_1}=1\\ {}{n}_1=\frac{\left({\sigma}_1^2+{\sigma}_2^2/K\right){\left({z}_{1-\alpha /2}+{z}_{1-\beta}\right)}^2}{\varDelta^2}\\ {}{n}_1=\frac{\left({6.6}^2+{6.6}^2/1\right){\left(1.96+0.84\right)}^2}{6.6^2}\\ {}{n}_1=16\\ {}{n}_2={K}^{\ast }{n}_1=16\end{array}}$$

- ∆ = |*μ*_2_ − *μ*_1_| = absolute difference between two means
- *σ*_1_, *σ*_2_ = variance of mean #1 and #2
- *n*_1_ = sample size for group #1
- *n*_2_ = sample size for group #2
- *α* = probability of type I error (usually 0.05)
- *β* = probability of type II error (usually 0.02)
- *z* = critical Z value for a given *α* or *β*
- *k* = ratio of sample size for group #2 to group #1

The values of mean and standard deviations were taken from the reference article [18].

### Randomization and allocation concealment mechanism

The study participants were divided into two groups: a control group that received MAD (*n*=20) and a test group that received CMOA (*n*=20). A randomization list was constructed by a computer. On the basis of successive enrolments, participants were assigned random numbers. The clinical site was contacted after confirmation of eligibility (subjects who met all inclusion criteria), and a centralized online randomization method (https://randomizer.at/) was used. Patients were randomly assigned to one of two arms: MAD or CMOA, utilizing block randomization.

### Masking

The double-blind masking was done, where neither the patient nor the investigators were revealed about the type of test group allocated to the patient. Authors from the Sleep Medicine Department, DMIMS (DU), created the allocation sequence, enrolled participants, and assigned people to therapies. The record remained in the hands of the the authors from Sleep Medicine Department who were not in direct contact with the patients and investigators.

### Inclusion criteria

1. The subjects suffering from moderate OSA diagnosed in PSG (AHI >5–15)
2. Patients non-compliant with CPAP
3. Patients not willing to a surgical intervention therapy for OSA
4. Between the ages of 30 and 50
5. Body mass index between 17 and 39 kg/m^2^

### Exclusion criteria

1. PSG revealing patients with severe OSA
2. Subjects who refuse to cooperate
3. Patients who have been diagnosed with advanced periodontitis
4. The arches are fully edentulous, or there are not enough teeth left in the arches to keep the appliance in place
5. Patients who have been diagnosed with temporomandibular joint problems
6. Patients who have been diagnosed with airway blockage
7. Patients having a maximum protrusion of less than 6 mm are referred to as “minimal protrusion” patients

### Calibration of examiner

For the training purpose, examiners were imparted a manual narrating the study protocols and examination criteria and directions concerning the examination of the subjects. Two examiners were selected each from the Department of Sleep Medicine for PSG reading, the Prosthodontic and Orthodontic departments for the fabrication of the interventions, and the experts for CAD-CAM designing.

### Intervention

#### Mandibular advancement device (MAD) (Figs. 2 and 3)

Twenty participants of group I (the control group) received MAD. The upper and lower arch impressions were recorded in irreversible hydrocolloid impression material (DPI Algitex) and the cast was poured in dental stone (Kalabhai). Using a George Gauge, the protrusion index was calculated for all study participants by extending the jaw to 60–80% of its maximal protrusion (roughly 6 mm). The upper and bottom halves of the device were made of acrylic, and they were joined by moving the mandible 6mm forward from its central location. Symptoms related to temporomandibular disorder (TMD) were assessed but none of the study group participants had any complaints related to it. All the patients were counseled to wear the appliance during sleeping for a minimum of 6 h daily.

**Fig. 2:**
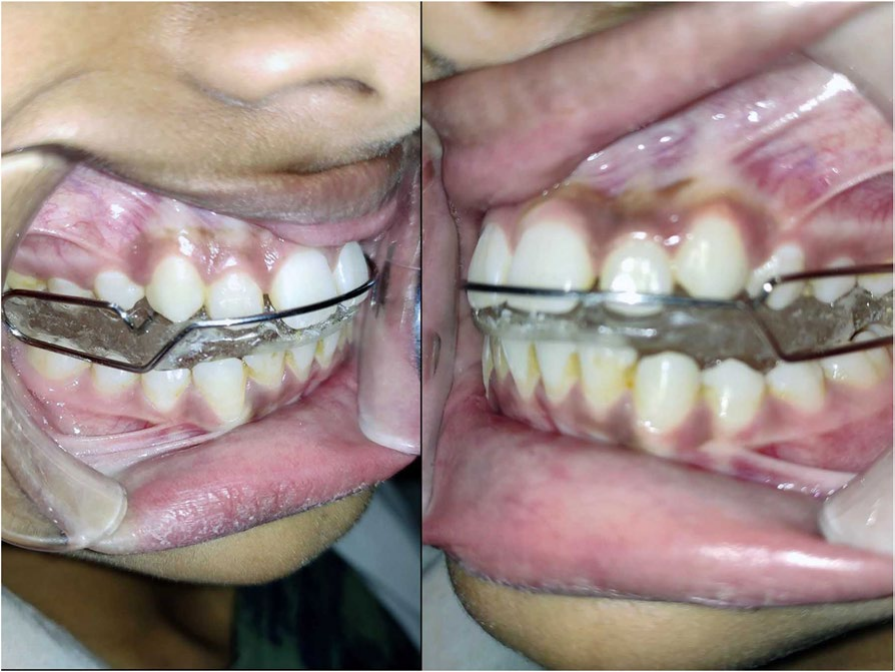
MAD placed intra-orally

**Fig. 3:**
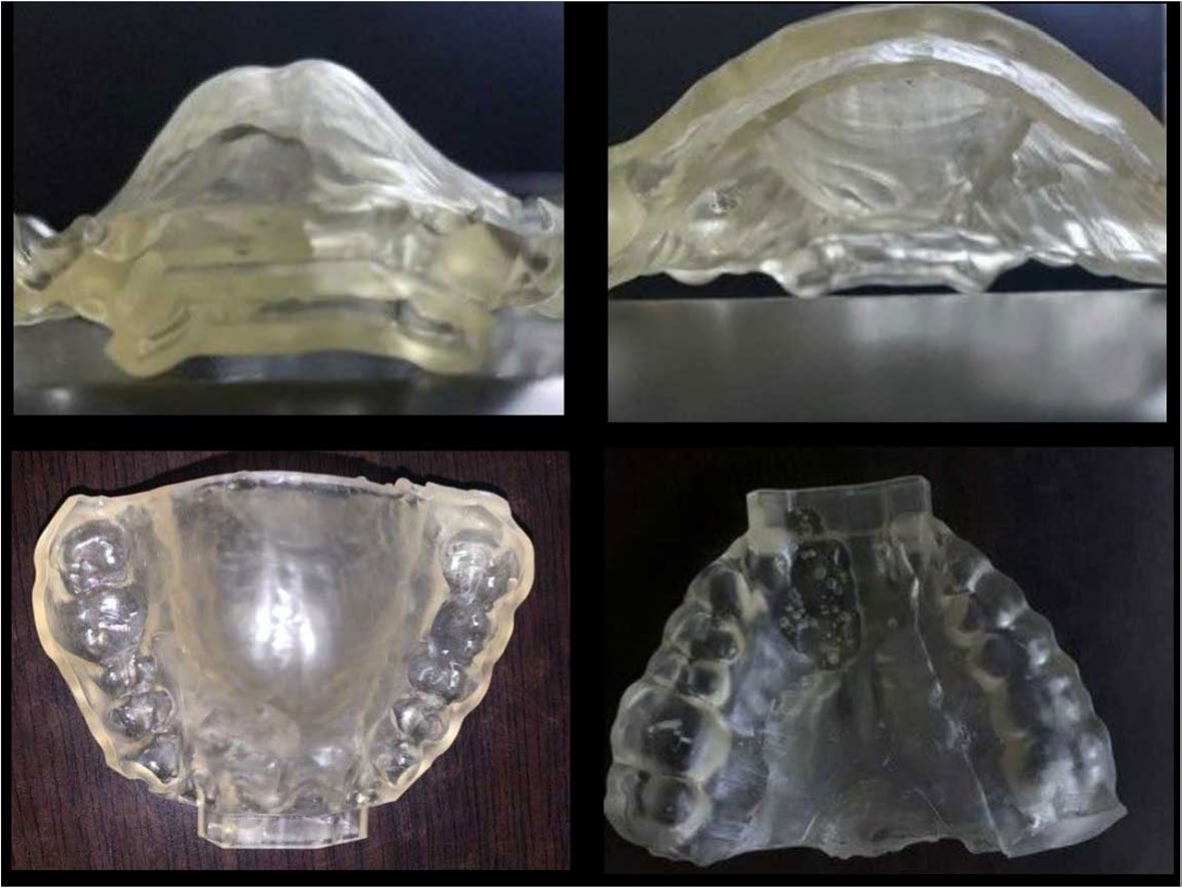
CMOA

#### Customized maxillary oral appliance (CMOA) (Figs. 4 and 5)

##### Design

CMOA is a customized maxillary removable oral appliance with a “base plate” and the “counter plate.” The base plate was adjusted over the upper jaw taking support of hard and soft tissues that is namely teeth and hard palate. The counter plate is adjusted over the base plate with a space of 2 mm in between the two plates. The space between these two plates was made empty or hollow. The upper plate had the anatomy of the occlusal surface of the upper teeth for occluding with the lower teeth in present occlusal relation but at an increased vertical dimension that is attained by hollowing the plate. The hollow upper plates had an opening or a hole in the central incisor area that is in the anterior region, for the uninterrupted instreaming of the fresh air towards the posteriors region of the tongue. Moreover, a bulge was outlined on the upper plate on the palatal aspect of the appliance in the most posterior back region to restrain the tongue from fallback.

**Fig. 4:**
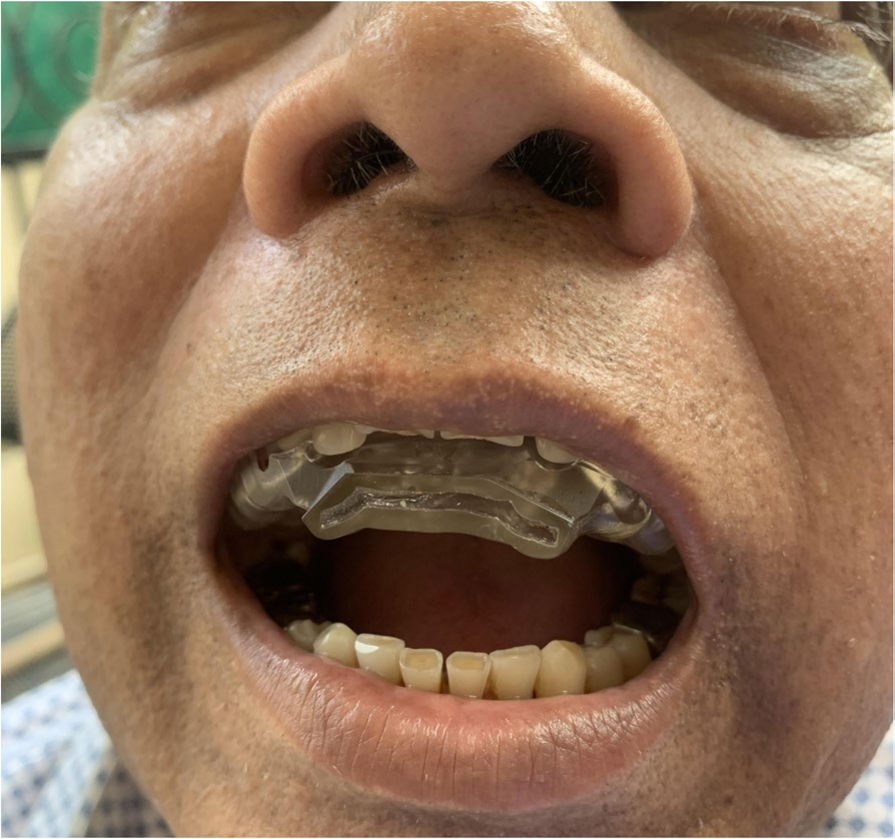
Frontal view of CMOA placed intra-orally

**Fig. 5:**
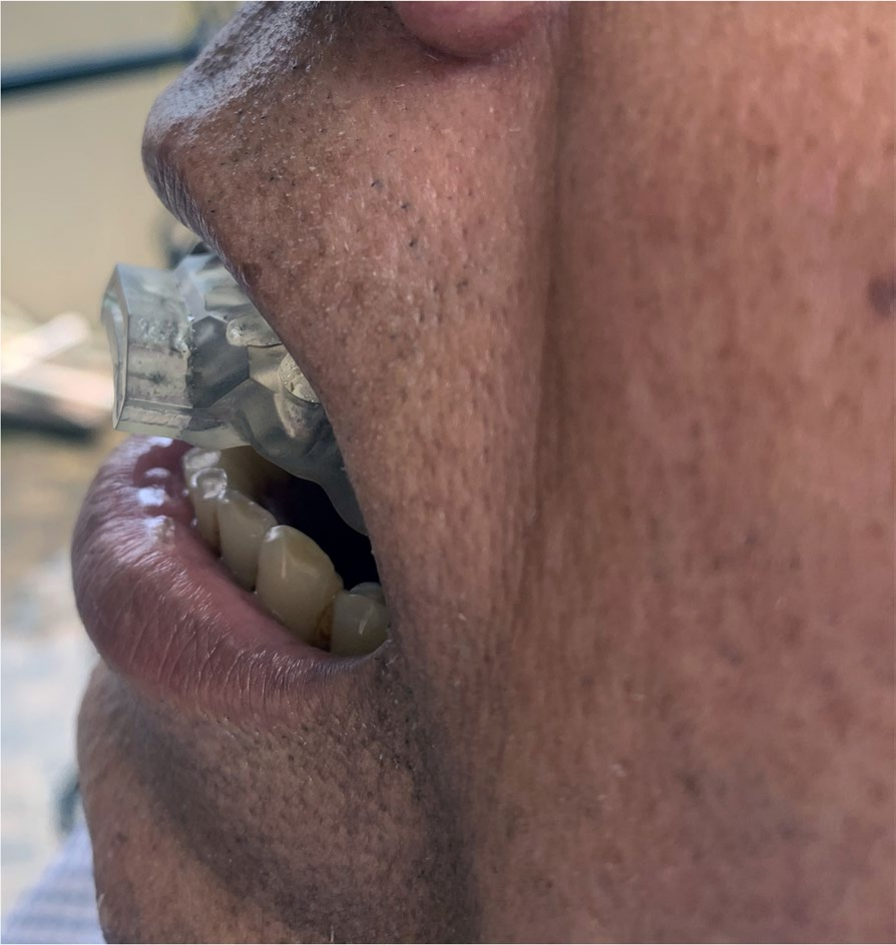
Lateral view of CMOA placed intra-orally

##### Fabrication of CMOA

The upper and lower arch were scanned (3shape TRIOS 4). Computer-aided designing (CAD) was done using CAD software. The design was then 3D printed in 3D printing epoxy resin material (eResin-PLA). This plate was customized for 20 participants of group II. All the patients were counseled to use the appliance during sleeping for a minimum of 6 h daily.

### Investigations

#### Polysomnography (PSG)

A nocturnal PSG (EMBLA(R)S7000, EmblaSystem, Inc., Broomfield, CO., USA) was done in the sleep medicine department to get references or baseline measures and after 1 month and 3 months of use of MAD and CMOA and the AHI was measured.

#### Oxygen saturation in the blood (SpO2)

Oxygen saturation in blood was calculated using a finger pulse oximeter (OTICA CONTEC CMS 5100) at the time of PSG, and its values were calculated for references and after 1 month and 3 months of use of MAD and CMOA. Mean oxygen saturation and the proportion of time with SpO2 <90% were assessed.

#### Oro-nasal airflow via a pressure transducer

A pressure transducer (OTICA CONTEC CMS 5100) was used to determine the mean respiratory disturbance index (RDI).

#### Epworth Sleepiness Scale (ESS)

ESS was applied for self-assessment of the level of sleepiness during the daytime.

### Safety evaluation

Any serious adverse events related to epoxy resin material sensitivity, TMJ pain, or muscle pain because of the rise in vertical occlusal measures, pharyngeal or gag reflex, or others were not found in any study participant.

### Statistical analysis

The reference measures that were recorded before delivering the appliance were compared with the values seen after a month and 3 months of appliance delivery. Descriptive and analytical statistics were performed. The values were presented in mean and standard deviations. The Shapiro-Wilk test was used to determine the normality of continuous data. Because the data had a normal distribution, parametric tests were used to investigate it. To corroborate the mean differences, the independent sample *t*-test and paired sample *t*-test were used. The significance threshold was maintained at *P*<0.05. The statistical program used was “SPSS (Statistical Package for Social Sciences) Version 20.1” (IBM Corporation, Chicago, USA).

## Result

The results of the nocturnal polysomnography are shown in Tables 1, 2, 3, 4, 5, 6, 7, and 8. The inter- and intragroup analysis of mean AHI, SPO2, RDI, and ESS of MAD and CMOA groups was done. The paired *t*-test applied for intragroup analysis showed that both the groups (MAD and CMOA) for all the parameters (AHI, SPO2, RDI, and ESS) had statistically significant values (*P*<0.001). When comparing reference measurements to 1-month measures, 1- to 3-month measures, and 3 months to reference measures, there was a statistically significant reduction in mean AHI scores, improvement in mean SPO2 scores, and reduction in mean RDI scores and ESS scores. The reduction in mean AHI score for MAD was from 22.80 to 14.00 events/h after 1 month and 10.60 events/h after 3 months and for CMOA was from 24.95 to 13.85 events/h after 1 month and 10.45 events/h after 3 months. The SpO2 nadir increased from 85.70 to 95.15% after 1 month and further to 97.20% after 3 months of intervention by MAD. CMOA showed increased SpO2 nadir from 84.95 to 94.70% after 1 month and further to 96.80% after 3 months. The reduction in mean RDI score for MAD was from 23.35 to 13.05 events/h after 1 month and 10.85 events/h after 3 months and for CMOA was from 22.95 to 12.80 events/h after 1 month and 10.65 events/h after 3 months. The mean score of ESS was 20.30 before treatment by MAD which reduced to 11.85 after 1 month and to 8.90 after 3 months with occasional snoring. All the values were statistically significant when compared to baseline values for both the test groups individually but when compared with each other both the groups did not show statistical significance.

**Table 1:** Intragroup comparison of the mean AHI of the MAD and CMOA groups

Groups	Timeline	***N***	Mean	S.D.	***P***-value^#^
**MAD**	**Baseline**	20	22.80	0.068	<0.001^†^
	**1 month**	20	14.00	1.71	
	**1 month**	20	14.00	1.71	<0.001^†^
	**3 months**	20	10.60	1.27	
	**Baseline**	20	22.80	0.720	<0.001^†^
	**3 months**	20	10.60	1.27	
**CMOA**	**Baseline**	20	24.95	3.56	<0.001^†^
	**1 month**	20	13.85	1.84	
	**1 month**	20	13.85	1.84	<0.001^†^
	**3 months**	20	10.45	1.35	
	**Baseline**	20	24.95	3.56	<0.001^†^
	**3 months**	20	10.45	1.35	

^#^*P*-value derived from independent sample *t*-test

^†^Significant at *P* < 0.05

**Table 2:** Intragroup comparison of mean SPO_2_ of the MAD and CMOA groups

Groups	Timeline	***N***	Mean %	S.D.	***P***-value^#^
**MAD**	**Baseline**	20	85.70	4.47	<0.001^†^
	**1 month**	20	95.15	1.18	
	**1 month**	20	95.15	1.18	<0.001^†^
	**3 months**	20	97.20	1.10	
	**Baseline**	20	85.70	4.47	<0.001^†^
	**3 months**	20	97.20	1.10	
**CMOA**	**Baseline**	20	84.95	4.94	<0.001^†^
	**1 month**	20	94.70	1.92	
	**1 month**	20	94.70	1.92	<0.001^†^
	**3 months**	20	96.80	1.23	
	**Baseline**	20	84.95	4.94	<0.001^†^
	**3 months**	20	96.80	1.23	

^#^*P*-value derived from independent sample *t*-test

^†^Significant at *P* < 0.05

**Table 3:** Intragroup comparison of mean RDI of the MAD and CMOA groups

Groups	Timeline	***N***	Mean	S.D.	***P***-value^#^
**MAD**	**Baseline**	20	23.35	2.64	<0.001^†^
	**1 month**	20	13.05	1.82	
	**1 month**	20	13.05	1.82	<0.001^†^
	**3 months**	20	10.85	1.30	
	**Baseline**	20	23.35	2.64	<0.001^†^
	**3 months**	20	10.85	1.30	
**CMOA**	**Baseline**	20	22.95	2.32	<0.001^†^
	**1 month**	20	12.80	1.85	
	**1 month**	20	12.80	1.85	<0.001^†^
	**3 months**	20	10.65	1.38	
	**Baseline**	20	22.95	2.32	<0.001^†^
	**3 months**	20	10.65	1.38	

^#^*P*-value derived from independent sample *t*-test

^†^Significant at *P* < 0.05

**Table 4:** Intragroup comparison of mean ESS of the MAD and CMOA groups

Groups	Timeline	***N***	Mean	S.D.	***P***-value^#^
**MAD**	**Baseline**	20	20.30	2.10	<0.001^†^
	**1 month**	20	11.85	1.34	
	**1 month**	20	11.85	1.34	<0.001^†^
	**3 months**	20	8.90	1.07	
	**Baseline**	20	20.30	2.10	<0.001^†^
	**3 months**	20	8.90	1.07	
**CMOA**	**Baseline**	20	19.75	2.65	<0.001^†^
	**1 month**	20	12.00	1.29	
	**1 month**	20	12.00	1.29	<0.001^†^
	**3 months**	20	9.05	0.99	
	**Baseline**	20	19.75	2.65	<0.001^†^
	**3 months**	20	9.05	0.99	

^#^*P*-value derived from independent sample *t*-test

^†^Significant at *P* < 0.05

**Table 5:** Intergroup comparison of the mean apnea-hypopnea index (AHI) between the two groups

Timeline	Groups	***N***	Mean	S.D.	***P***-value^#^
**Baseline**	**MAD**	20	22.80	3.67	0.068
	**CMOA**	20	24.95	3.56	
**1 month**	**MAD**	20	14.00	1.71	0.791
	**CMOA**	20	13.85	1.84	
**3 months**	**MAD**	20	10.60	1.27	0.720
	**CMOA**	20	10.45	1.35	

^#^*P*-value derived from independent sample *t*-test

**Table 6:** Intergroup comparison of mean SPO_2_ between the two groups

Timeline	Groups	***N***	Mean	S.D.	***P***-value^#^
**Baseline**	**MAD**	20	85.70	4.47	0.618
	**CMOA**	20	84.95	4.94	
**1 month**	**MAD**	20	95.15	1.18	0.378
	**CMOA**	20	94.70	1.92	
**3 months**	**MAD**	20	97.20	1.10	0.288
	**CMOA**	20	96.80	1.23	

^#^*P*-value derived from independent sample *t*-test

**Table 7:** Intergroup comparison of mean respiratory disturbance index (RDI) between the two groups

Timeline	Groups	***N***	Mean	S.D.	***P***-value^#^
**Baseline**	**MAD**	20	23.35	2.64	0.614
	**CMOA**	20	22.95	2.32	
**1 month**	**MAD**	20	13.05	1.82	0.669
	**CMOA**	20	12.80	1.85	
**3 months**	**MAD**	20	10.85	1.30	0.642
	**CMOA**	20	10.65	1.38	

^#^*P*-value derived from independent sample *t*-test

**Table 8:** Intergroup comparison of mean Epworth Sleepiness Scale (ESS) between the two groups

Timeline	Groups	***N***	Mean	S.D.	***P***-value^#^
**Baseline**	**MAD**	20	20.30	2.10	0.472
	**CMOA**	20	19.75	2.65	
**1 month**	**MAD**	20	11.85	1.34	0.722
	**CMOA**	20	12.00	1.29	
**3 months**	**MAD**	20	8.90	1.07	0.650
	**CMOA**	20	9.05	0.99	

^#^*P*-value derived from independent sample *t*-test

## Discussion

When compared to the baseline measurements, the results of the present study showed a significant improvement in the AHI due to a decrease in both groups’ apnea and hypopnea events. In comparison to the results from the 1-month follow-up measurements, the 3-month follow-up measurements revealed a more pronounced decline in the values and events. Similarly, SpO2, RDI, and the ESS also showed a significant increase in values indicating improvement after intervention by MAD and CMOA.

The standard therapeutic approaches for OSA are CPAP and OA [19]. Zhang et al. and Schwartz et al. in 2019 and 2018 respectively executed a meta-analysis to analyze the efficacy of OA against CPAP to manage OSA. Their study results concluded that CPAP had better efficacy in lowering the AHI score; at the same time, it had notably lower compliance that nullified the difference created by the score against MAD in terms of quality of life and cognitive outcomes [20, 21].

Some OAs are available in the market and even recorded in the literature for treating “mild to moderate” OSA, with MAD having the most successful and recommended results [22, 23].

The goal of this study was to see if the CMOA may be a good oral appliance for people with moderate OSA. The findings of this study confirmed the study’s premise by demonstrating statistically significant differences between the reference measures of all measured parameters and the measures collected after a month and after 3 months of MAD and CMOA delivery. However, statistical analysis of the acquired data revealed no significant variations in measured values between the MAD- and CMOA-treated groups, confirming the efficacy of CMOA in controlling OSA.

The CMOA increases the vertical dimension by 2mm, which results in the advanced and descending position of the lower jaw which in turn increases the flow of air by keeping the patency of the airway maintained. The vertical dimension of occlusion loss is observed in the population between the ages of 40 and 50 years; this appliance can aid in regaining the vertical dimension and also in re-establishing the actual centric relation [24]. Along with OSA, these appliances can also be used in treating the signs and symptoms of temporomandibular disorders (TMD) that are present because of vertical dimension loss. Within the period of study, CMOA did not cause any alteration in dentition as noticed in patients who received MAD which is the major advantage of using CMOA over MAD, making it more compliant among patients. However, long-term studies are required to prove these facts.

Cardinal features of the CMOA are:

1. Backfall of the tongue was prevented by the bulge designed over the palate of the appliance
2. The constant influx of fresh air was facilitated by the hole provided in the anterior region of the appliance
3. Airflow was directed towards the pharynx by making the appliance hollow
4. The precision was maintained in designing and manufacturing by the use of CAD-CAM

In both devices, the vertical dimension is increased by 2–4 mm depending on the patient’s tolerance and comfort. However, in MAD, this increase in vertical dimension is done by not only opening the bite but also by forwarding the mandible. This causes rotational as well as translational movement resulting in the remodeling of the temporomandibular joint in a new unfavorable position, whereas the CMOA rotates the condyle by opening the bite at existing occlusal relation without or with the minimal translational movement of the joint. So there will be hardly any occlusal disharmony in CMOA.

Before and after the intervention, both study groups underwent a PSG, which is the benchmark in diagnosing and grading OSA. Hypopnea (50% or less than 50% reduction in airflow) and obstructive apnea (10-s cessation of airflow) events were observed [25]. The results revealed significant improvement in the AHI by the reduction in the events of apnea and hypopnea in both groups when compared to the baseline measures. Three-month follow-up measurements showed a more significant reduction in the values and events as compared to the 1-month follow-up results. Alike results were reported by Guimaraes et al. in 2018 [26]. They found improved results in the AHI from 80.5 to 14.6 events/h after successful MAD therapy. Basyuni has stated in their article which was an update on MAD as a therapy for obstructive sleep apnea syndrome that studies since 2005 on managing OSA with MAD have revealed a reduction in mean AHI between 30 and 72% [27].

According to Otero et al., the AHI alone is insufficient to rate the severity of OSA [28]. As a result, in addition to AHI, SPO2, oro-nasal airflow, and ESS were also assessed in the current study to track changes in the severity of OSA before and after 1 and 3 months of MAD and CMOA treatments.

MAD is strongly connected with poor nocturnal blood oxygenation in patients suffering from severe OSA, and as a result, it is a suggested predictor of blood oxygenation. Because nocturnal hypoxia is connected to OSA morbidity and death, SpO2 is thought to have predictive value. The data obtained from the present study have shown a significant increase in SPO2 values after the intervention by MAD and CMOA. The current study’s findings are consistent with those of Fietze et al. and Temirbekov et al., who investigated the oxygen desaturation index (ODI) [29, 30]. The present study result can be an evidence base document to establish a correlation between SpO2 and AHI in OSA patients.

The fluctuation in nasal pressure was detected using a nasal transducer. The episodes of the AHI/hour of total sleep time is the RDI. Both test groups showed a notable reduction in the values of RDI from the baseline measures. Similar results were seen in the study piloted by Rose et al., Gauthier et al., Blanco et al., Hans et al., and Lawton et al. [31–35].

Murray Johns introduced ESS in early 1991 to assess daytime sleepiness, and it has been linked to OSA [36]. It is a numerical scale where a score of more than 10 indicates the presence of sleepiness. This scale has been used in numerous research to diagnose and measure therapy outcomes [37, 38]. The data obtained from the present study showed the ESS score ranging from 13 to 15. And the score was highest for the question about sitting quietly after lunch followed by watching TV. When administered sequentially, the ESS scores varied, which must be attributable to the subjective nature of the study. However, the data obtained after 3 months of intervention showed a drastic reduction in the ESS score when compared to the baseline score. But the value was not significant between the MAD and CMOA test groups.

Despite the fact that oral appliances can be employed in a variety of OSA patients, they have a number of limitations, including the absence of enough teeth in the maxillary and mandibular arches. A tooth is thought to be especially crucial for ensuring the stability and retention of the mandibular advancement device. The condition of edentulism inherently exacerbates OSA and limits the number of viable therapies [39]. However, a review of the literature finds that only a few papers describe the use of MAD in the treatment of edentulous patients or patients with multiple missing teeth with OSA [40]. Some modifications are a must in the basic design of MAD in such patients. But CMOA can be successfully used not only in patients with fixed but also patients rehabilitated with a removable partial prosthesis as it takes major retention from the palatal slopes as does the removable partial dentures.

### Limitations of the present study

The study had a small sample size. which is a major limitation of the current study. Longitudinal studies involving other parameters such as rapid eye movement, non-rapid eye movement, electrocardiography, electroencephalogram, and oxygen desaturation index also need to be evaluated to prove the authenticity of the CMOA.

### Further scope

The CPAP machine can be easily attached to the CMOA with the help of a small connector. This can be effective and proven effective in managing severe OSA and will have a higher compliance rate as the need for a mask will be eliminated and replaced by the CMOA. To assess the utility of this hybrid architecture, more research is required (CMOA and CPAP). The appliance is being tested to see if it can effectively manage COVID-19 patients.

## Conclusion

To stratify such a treatment technique, a single tool ought to be avoided. And hence together with AHI, SPO2, oro-nasal airflow, and ESS were checked to corroborate the findings before introducing this novel design of custom-made maxillary oral appliances in the field of sleep medicine for the treatment of moderate OSA. Based on the findings of this study, it can be inferred that CMOA is as effective as MAD in treating moderate OSA.

## Data Availability

The data will be as per the request.

## Abbreviations

OA: Oral appliances
OSA: Obstructive sleep apnea
CPAP: Continuous positive airway pressure
RCT: Randomized controlled trial
AHI: Apnea/hypopnea index
MAD: Mandibular advancement device
CMOA: Customized maxillary oral appliance
TMD: Temporomandibular disorder
BMI: Basal metabolic index
RDI: Respiratory disturbance index
ESS: Epworth Sleepiness Scale
SpO_2_: Oxygen saturation in the blood
PSG: Polysomnography
PMMA: Polymethylmethacrylate
ODI: Oxygen desaturation index
3D: 3 dimensions
CAD-CAM: Computer-aided designing-computer-aided manufacturing
